# Synapsin IIb as a functional marker of submissive behavior

**DOI:** 10.1038/srep10287

**Published:** 2015-05-22

**Authors:** Elimelech Nesher, Igor Koman, Moshe Gross, Tatiana Tikhonov, Maryia Bairachnaya, Mali Salmon-Divon, Yishai Levin, Gabi Gerlitz, Izhak Michaelevski, Gal Yadid, Albert Pinhasov

**Affiliations:** 1 Department of Molecular Biology. Ariel University, Ariel. Israel; 2 Faculty of Life Sciences. Bar-Ilan University, Ramat Gan. Israel; 3 de Botton Institute for Protein Profiling, The Nancy and Stephen Grand Israel National Center for Personalized Medicine. Weizmann Institute of Science, Rehovot. Israel; 4 Department of Biochemistry and Molecular Biology. Tel-Aviv University, Tel-Aviv. Israel; 5 Sagol School of Neuroscience, Tel-Aviv University, Tel-Aviv. Israel

## Abstract

Dominance and submissiveness are important functional elements of the social hierarchy. By employing selective breeding based on a social interaction test, we developed mice with strong and stable, inheritable features of dominance and submissiveness. In order to identify candidate genes responsible for dominant and submissive behavior, we applied transcriptomic and proteomic studies supported by molecular, behavioral and pharmacological approaches. We clearly show here that the expression of Synapsin II isoform b (Syn IIb) is constitutively upregulated in the hippocampus and striatum of submissive mice in comparison to their dominant and wild type counterparts. Moreover, the reduction of submissive behavior achieved after mating and delivery was accompanied by a marked reduction of Syn IIb expression. Since submissiveness has been shown to be associated with depressive-like behavior, we applied acute SSRI (Paroxetine) treatment to reduce submissiveness in studied mice. We found that reduction of submissive behavior evoked by Paroxetine was paired with significantly decreased Syn IIb expression. In conclusion, our findings indicate that submissiveness, known to be an important element of depressive-like behavioral abnormalities, is strongly linked with changes in Syn IIb expression.

Interactions among members of a given animal community are governed by each individuals’ standing in the social hierarchy^1^,^2^. Based on the competition for territory, food and mating partner, two opposite behavioral types can be described: dominance and submissiveness^2^,^3^,^4^. In any interaction between two individuals of the same species, one will demonstrate features of dominance while the other will adopt a submissive posture^5^. It was suggested that dysfunctional expressions of dominance and submissiveness in humans can be linked to the etiology of various pathological conditions such as personality disorders, neurodegenerative diseases^6^,^7^,^8^, mania and depression^9^,^10^. To study behavioral and regulatory aspects of dominance and submissiveness, we employed a mouse model demonstrating strong and stable inheritable features of dominance and submissiveness^2^,^11^. These dominant and submissive mice react differentially to stress-inducing factors, antidepressants and mood stabilizing agents and their inherited behavioral tendencies were shown to be also dependent upon environmental and social triggers^2^,^11^,^12^,^13^.

Recent studies demonstrated that the regulation of social behavior involves synaptic genes^14^,^15^, particularly the Synapsin family of genes (Syn I, Syn II, Syn III)^15^, which may be alternatively spliced into ten different isoforms (a and b for Syn I and II, and a-f for Syn III)^16^. The main function of these proteins is the modulation of neurotransmitter release at the pre-synaptic terminal by reversibly tethering synaptic vesicles (SVs) to the actin cytoskeleton^16^,^17^,^18^. Synapsins are believed to act in concert with other synaptic genes, and their expression has been shown to correlate with that of other genes involved in synaptic activity (Rab3a, SV2a, Syp and others)^17^,^19^. Both pre-clinical and clinical studies showed that Synapsins may be implicated in neuropsychiatric disorders, including bipolar disorder^20^, schizophrenia^21^,^22^, autism^15^,^18^ and epilepsy^15^,^17^,^23^. Targeted deletion of Synapsin genes leads to cognitive impairments^15^,^24^, behavioral abnormalities and deficits in social interaction^15^,^22^.

Thus, we hypothesized that the distinct behavior of selectively bred dominant and submissive mice may correlate with changes in synaptic activity. By studying Synapsin genes via transcriptomic and proteomic analyses, we found for the first time a strong link between Syn IIb expression and submissive behavior in mice, and focused further study of this relationship using molecular, behavioral and pharmacological approaches.

## Results

### Synapsin IIb isoform is markedly upregulated in Submissive mice

**Figure 1:**
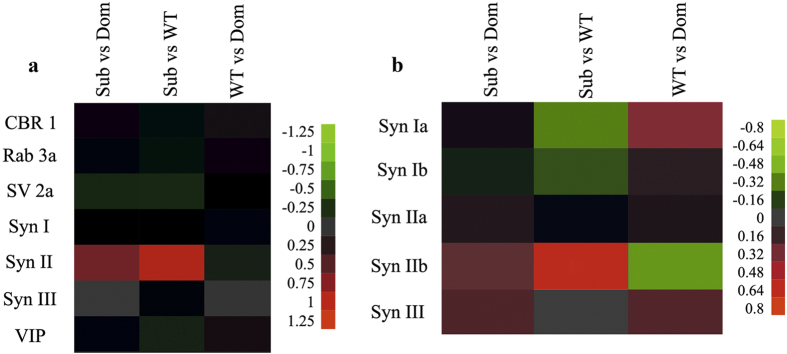
Correlation between hippocampal mRNA and protein levels of differentially expressed Synapsins. **** Heatplot representation of log_2_ fold changes in gene expression between mouse groups detected by microarray (**a**) and proteomic (**b**) analysis with red and green representing an increase or decrease in fold expression, respectively. Presented data was extracted from microarray and proteomic preprocessing, subjected to hierarchical clustering (Euclidean and Pearson correlative metrices for proteomic and microarray analysis, respectively). Data assembly was performed using Ward’s and single linkage for proteomic and microarray data, respectively, followed by functional enrichment using gene ontology clustering implemented in the Database for Annotation, Visualization and Integrated Discovery (DAVID) v6.7^56^ bioinformatics tool.

**Figure 2:**
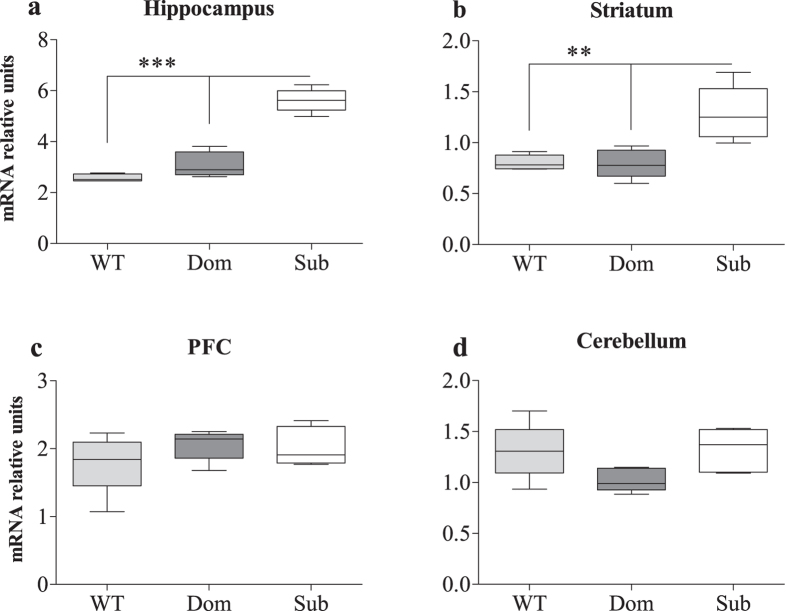
The Syn IIb isoform is markedly upregulated in the hippocampus and striatum of Submissive animals. **** mRNA levels in the hippocampus and striatum show significant upregulation of Syn IIb (**a** and **b**) among submissive (Sub) mice, in comparison to their dominant (Dom) and wild type (WT) counterparts. No differences were found in expression of the Syn IIb isoform in the prefrontal cortex (PFC) and cerebellum (**c** and **d**) of tested mice. The statistical significance between animal groups was assessed using one way ANOVA, followed by Bonferroni post hoc analysis, indicated by (**) at *p* < 0.01 and (***) at *p* < 0.001; n = 5 for each group.

Microarray analysis of mRNA extracted from hippocampi of submissive (Sub), dominant (Dom) and wild type (WT) mice followed by qRT-PCR validation revealed changes in the expression of the Synapsin (Syn) II gene (Fig. 1a). Since Syn II has two active isoforms, we checked whether the changes in Syn II mRNA levels in submissive animals are specific to one of the isoforms or are common to both of them. One way ANOVA analysis revealed significant differences in hippocampal Syn IIb expression among Sub, Dom and WT (Fig. 2a; *F*(2, 12) = 85.73, *p* < 0.0001). Further Bonferroni post hoc multiple comparison test indicated significantly upregulated levels of Syn IIb in the hippocampus of Sub mice in comparison to both WT and Dom animals (Fig. 2a; *t* = 12.24, *p* < 0.001 and *t* = 10.15, *p* < 0.001 respectively). We did not find significant differences in hippocampal Syn IIb expression between Dom and WT mice (Fig. 2a; *t* = 2.09, ns). The same Syn IIb expression pattern was also observed in the striatum (Fig. 2b; *F*(2, 12) = 12.39, *p* = 0.0012; *t* = 4.26, *p* < 0.01 for Sub vs WT; *t* = 4.36, *p* < 0.01 for Sub vs Dom; *t* = 0.10, ns for WT vs Dom), while Syn IIb expression in the prefrontal cortex (PFC) (Fig. 2c; *F*(2, 12) = 1.05, *p* = 0.3810) and cerebellum (Fig. 2d; *F*(2, 12) = 3.18, *p* = 0.0779) showed no differences.

**Figure 3:**
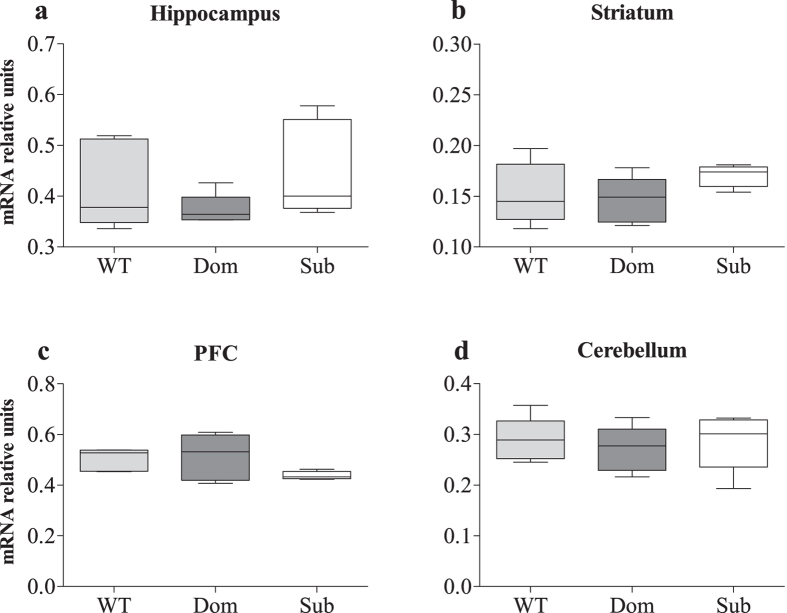
The Syn IIa isoform showed no differences in the analyzed brain parts of Submissive animals. **** No differences were found in expression of the Syn IIa isoform in the hippocampus, striatum, prefrontal cortex (PFC) and cerebellum of tested mice. n = 5 for each group.

At the same time, one way ANOVA analysis did not reveal significantly different Syn IIa expression in the hippocampus (Fig. 3a; *F*(2, 12) = 1.33, *p* = 0.3020), striatum (Fig. 3b; *F*(2, 12) = 1.50, *p* = 0.2623), PFC (Fig. 3c; *F*(2, 12) = 2.38, *p* = 0.1349) and cerebellum (Fig. 3d; *F*(2, 12) = 0.20, *p* = 0.8208) among all tested groups.

**Figure 4:**
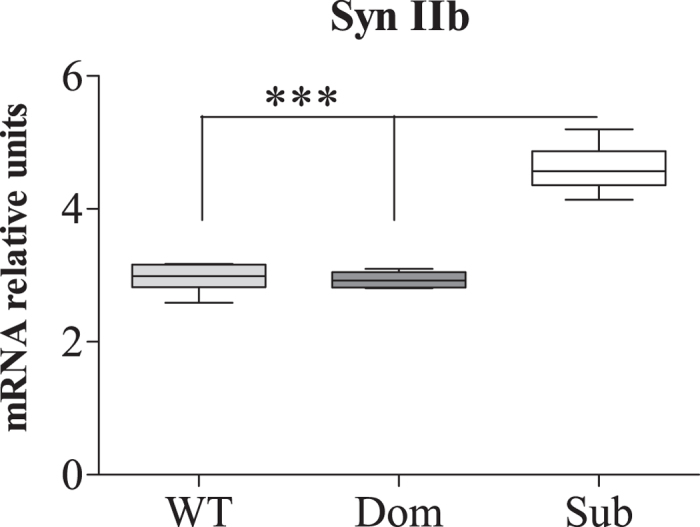
Syn IIb isoform is markedly upregulated in the hippocampus of Submissive mice at birth. **** Syn IIb levels were strongly upregulated in Sub pups on postnatal day 1. The statistical significance between animal groups was assessed using one way ANOVA, followed by Bonferroni post hoc analysis, indicated by (***) at *p* < 0.001; n = 6 for each group.

**Figure 5:**
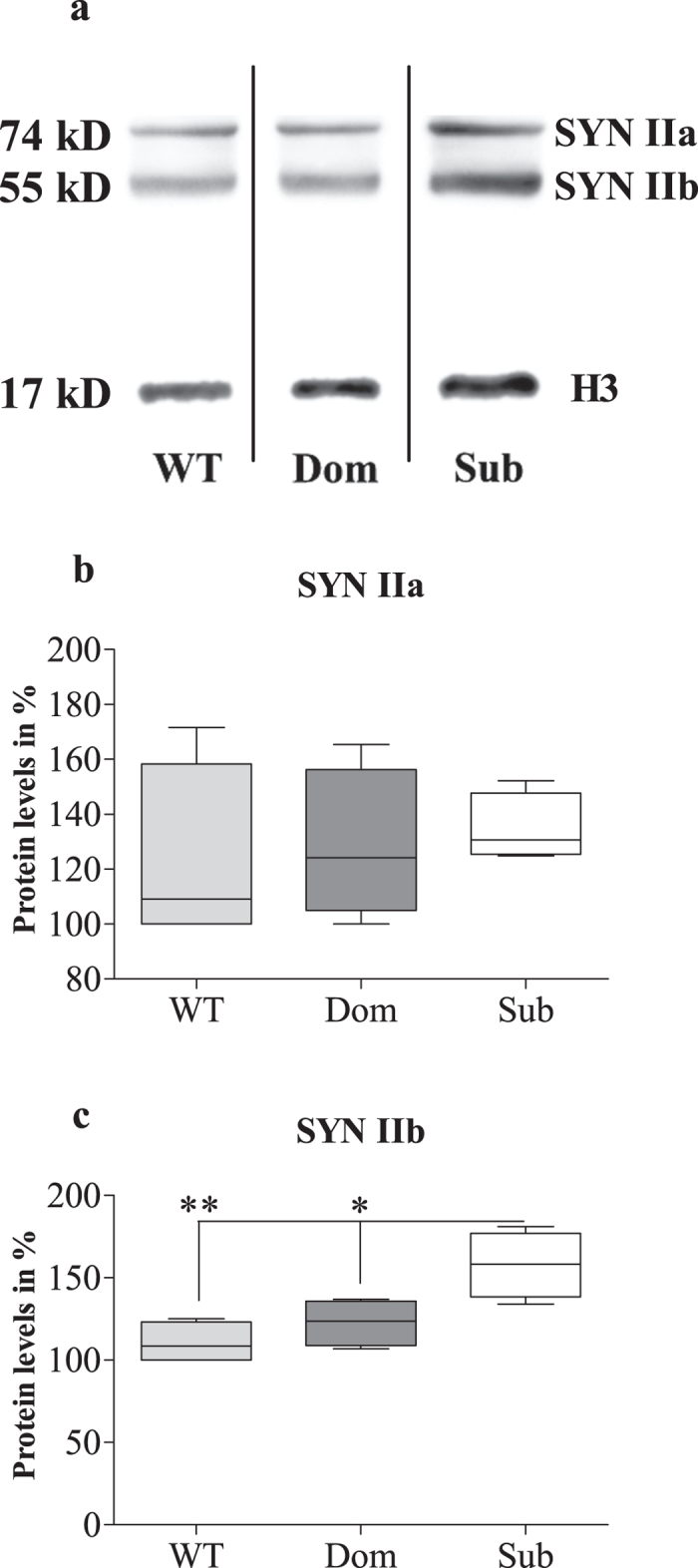
Quantification of SYN IIa and SYN IIb protein levels in the hippocampus of wild type (WT), dominant (Dom) and submissive (Sub) mice. Representative immunoblots (**a**) demonstrate the high SYN IIa protein levels in Sub mice. Protein blots of mice from three different groups (WT, Dom and Sub) were cropped from the same membrane. SYN IIa protein expression showed no differences among all tested groups (**b**). SYN IIb protein levels were observed to be significantly upregulated among Sub mice compared to their Dom and WT counterparts (**c**). Molecular weight is labelled in kD, with histone H3 used as a loading control. Data are expressed as the mean SEM, after the average value of WT was set to 100%. Statistical significance between groups was assessed using one way ANOVA, followed by Bonferroni post hoc analysis, indicated by (*) at *p* < 0.05 and (**) at *p* < 0.01; n = 4 for each group.

We found it important to highlight that Syn IIb level is strongly upregulated not only in adult mice (Fig. 3a), but also in the hippocampus of Sub pups on postnatal day (PND) 1 (Fig. 4; one way ANOVA: *F*(2, 15) = 87, *p* < 0.0001; Bonferroni post hoc analysis: *t* = 11.31, *p* < 0.001 for Sub vs WT and *t* = 11.53, *p* < 0.001 for Sub vs Dom; WT vs Dom was not significant, *t* = 0.22), further highlighting the inherited basis of submissive behavior, as we have shown previously^2^. High throughput proteomic data (Fig. 1b) comparing protein expression levels of Dom, Sub and WT mice correlated well with the outcome of Syn IIb mRNA expression analysis in hippocampus. Thus, proteomic analysis revealed SYN IIb protein levels to be increased significantly in Sub mice vs WT animals (one sample two tailed *t*-test: *t* = 28,919, *p* < 0.01). Proteomic data was validated using immunoblot analysis showing enhanced protein expression of SYN IIb in hippocampus among Sub mice in comparison to WT and Dom groups (Fig. 5a,c; one way ANOVA: *F*(2, 9) = 9.55, *p* = 0.006; Bonferroni post hoc analysis: *t* = 4.21, *p* < 0.01 for Sub vs WT and *t* = 3.13, *p* < 0.05 for Sub vs Dom; WT vs Dom was not significant, *t* = 1.08), while SYN IIa protein expression showed no differences among all tested groups (Fig. 5a,b; one way ANOVA: *F*(2, 9) = 0.2134, *p* = 08118).

### Parturition markedly reduced Synapsin IIb expression and attenuated submissive behavior of Submissive dams

**Figure 6:**
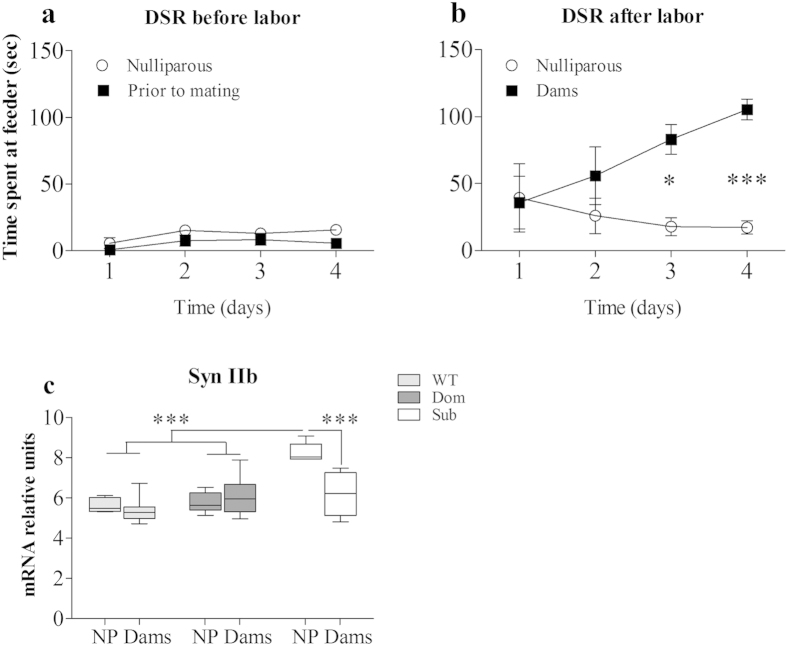
Parturition markedly reduced Syn IIb expression and attenuated submissive behavior of Submissive dams. **** Delivery accompanied a reduction of submissive behavior in submissive (Sub) dams, compared to their behavior prior to delivery (**a**) and to that of nulliparous (NP) females (**b**). Syn IIb levels also significantly decreased in the hippocampi of Sub Dams relative to Sub NP mice, reaching those of wild type (WT) and dominant (Dom) females (**c**). The statistical significance between animal groups was assessed using one- and two-way ANOVA with post-hoc Bonferroni test, indicated by (*) at *p* < 0.05 and (***) at *p* < 0.001; n = 7 for each group.

Parturition and maternal care are pivotal social events eliciting hormonal responses, including elevated release of Oxytocin, which is strongly implicated in social interactions^25^. Furthermore, studies demonstrated that dams display elevated defensive behavior postpartum^26^,^27^. Thus, we anticipated that postpartum Sub dams would demonstrate attenuation of their submissive behavior, accompanied by reduced Syn IIb expression. To test this hypothesis, we aimed to measure changes in social rank of Sub dams against nulliparous (NP). It was previously shown that lactating mice demonstrate increased aggression against female intruders in the Resident-Intruder test^28^, specifically based on a struggle over territory and designed to assess aggressive behavior^29^. Exposure of unmated submissive females to aggressive attacks of their counterparts was anticipated to lead to poor outcome of subsequent breeding and parturition, introducing artifacts to experimental results obtained. Thus, we used the DSR test to determine the social rank of mice prior to breeding, in which animals establish their social relationships without visible aggression. This approach allows us to avoid the exposure of unmated females to aggressive attacks of their counterparts. All females underwent the DSR test twice: prior to mating (Fig. 6a) and during the first postpartum week (Fig. 6b). We found that parturition accompanied a marked reduction of submissive behavior in Sub dams (Fig. 6b; two way ANOVA: *F*(1, 12) = 6.18, *p* = 0.0286; Bonferroni post hoc analysis: *t* = 2.97, *p* < 0.05 for the day 3 and *t* = 4.01, *p* < 0.001 for the day 4). Furthermore, as demonstrated in fig. 6c, hippocampal Syn IIb expression of Sub dams measured on day 8 after delivery was significantly reduced relative to NP mice (Fig. 6; one way ANOVA: *F*(5, 36) = 15.17, *p* < 0.0001; Bonferroni post hoc analysis: *t* = 5.59, *p* < 0.001), reaching the levels of WT and Dom females. Thus, we could identify a correlation between the reduction of submissiveness and decrease in Syn IIb expression among Sub dams.

### Mating markedly reduced Synapsin IIb expression and attenuated submissive behavior of Submissive males

**Figure 7:**
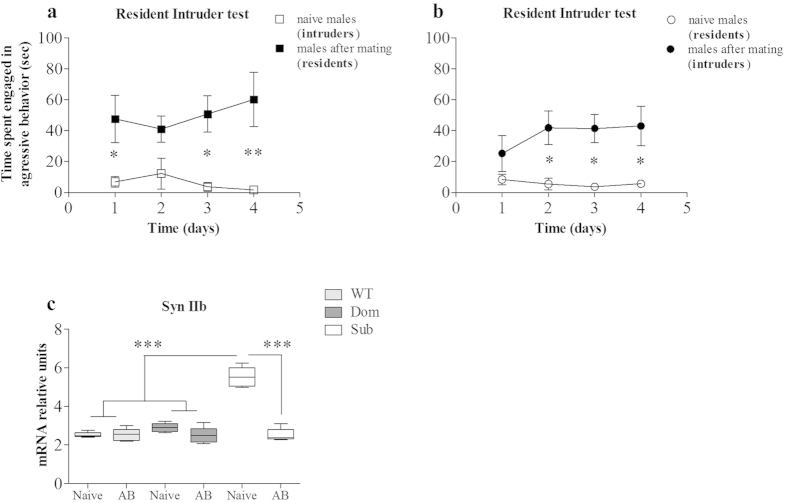
Mating markedly reduced Syn IIb expression and attenuated submissive behavior of Submissive males. **** The Resident-Intruder test demonstrated a reduction of submissive behavior in submissive (Sub) males after breeding (“AB”) compared to their naïve Sub counterparts (**a**, **b**). Syn IIb levels also significantly decreased in Sub males after mating relative to naive Sub males, reaching those of wild type (WT) and dominant (Dom) mice (**c**). The statistical significance between animal groups was assessed using one- and two-way ANOVA with post-hoc Bonferroni test, indicated by (*) at *p* < 0.05, (**) at *p* < 0.01 and (***) at *p* < 0.001; n = 5 for each group.

Mating behavior in mammals involves intricate social dynamics, often accompanied by fighting and aggression^30^,^31^, which are regulated by distinct molecular pathways^32^,^33^. Competition for a mating partner plays an important role in regulating males’ aggressive behavior both prior to^34^ and following^35^,^36^ intercourse. We anticipated that mating, in absence of a male competitor, would attenuate Sub males’ submissiveness. To investigate this hypothesis, we measured the Syn IIb expression levels of Sub males after mating in comparison to those of naïve Sub males, and found that mating reduced their hippocampal Syn IIb expression (Fig. 7c; one way ANOVA: *F*(5, 24) = 60.44, *p* < 0.0001; Bonferroni post hoc analysis: *t* = 13.72, *p* < 0.001), accompanied by a reduction in submissive behavior (Fig. 7a; two way ANOVA: *F*(1, 8) = 16.72, *p* = 0.0035; Bonferroni post hoc analysis: *t* = 2.75, *p* < 0.05, *t* = 3.18, *p* < 0.05 and *t* = 3.95, *p* < 0.01 for the days 1, 3 and 4 respectively. Fig. 7b; *F*(1, 8) = 26.54, *p* = 0.0009; Bonferroni post hoc analysis: *t* = 3.16, *p* < 0.05, *t* = 3.28, *p* < 0.05 and *t* = 3.24, *p* < 0.05 for the days 2, 3 and 4 respectively).

### Paroxetine dose-dependently downregulated Synapsin IIb expression of Submissive mice

**Figure 8:**
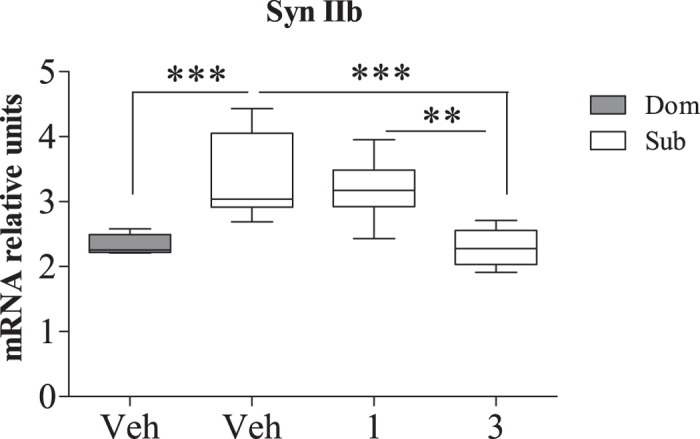
Paroxetine administration dose-dependently downregulated Syn IIb expression of Submissive mice. **** 3 mg/kg paroxetine reduced submissive mice’s Syn IIb levels, reaching those of vehicle-treated dominant animals. The statistical significance between animal groups was assessed using one-way ANOVA with post-hoc Bonferroni test, indicated by and (***) at *p* < 0.001; n = 8 for each group.

We previously demonstrated that the selective serotonin reuptake inhibitor (SSRI) paroxetine dose- dependently reduced immobility of Sub mice in the forced swim test (FST)^2^, a gold-standard measure of depressive-like phenotype. Thus, since submissiveness is associated with depressive-like behavior^3^,^13^, we speculated that paroxetine-driven reduction in depressive-like behavior may be associated with changes in Syn IIb expression. Indeed, we found that the antidepressive-like effects of paroxetine upon Sub mice was accompanied by significantly downregulated Syn IIb expression in the hippocampus, reaching the levels of Dom vehicle-treated animals (Fig. 8; one way ANOVA: *F*(3, 28) = 13.07, *p* < 0.0001; Bonferroni post hoc analysis: *t* = 4.95, *p* < 0.001). Taken together, these findings suggest that the expression level of Syn IIb can serve as a marker for submissiveness.

## Discussion

Social interactions play a fundamental role in determination of the quality of life, although they may be strong trigger of mental disorders^37^,^38^. In this study, integrative use of transcriptomic and proteomic strategies to identify candidate genes responsible for the expression of dominance (Dom) and submissiveness (Sub) led to the Synapsin II isoform b (Syn IIb) gene, member of the Synapsin family. By employing molecular, behavioral and pharmacological approaches, we examined the role of Syn IIb, using a mouse model demonstrating strong features of dominance and submissiveness – opposite forms of social behavior^2^,^11^. We show here for the first time, a significant link between Syn IIb isoform expression levels in the hippocampus and striatum and dominant and submissive behavior.

It was previously demonstrated that the hippocampus and striatum are involved in the regulation of social interactions^39^,^40^. Bidirectional synaptic plasticity between hippocampus and the striatum relies on activity of interneurons and contribute to the modulation of behavioral output^39^,^41^,^42^. Thus, both the hippocampus and striatum are deeply involved in the decision making^41^ believed to impact behavior status. Consequently, symptoms associated with antisocial behavior^43^, disrupted neurodevelopmental processes^44^, retrieval of emotional memories and contextual fear conditioning^45^ can be modulated by changes in neural networks connecting these regions. Moreover, disruption of different components of the hippocampus and striatum, may lead to various psychiatric and neurological disorders^46^. Since Sub mice display elevated levels of Syn IIb in the hippocampus and striatum (Fig. 2), we hypothesized that reduction in Sub mice’s submissive behavior should accompany reduction in Syn IIb expression. To assess this hypothesis, we took advantage of the known phenomena that female’s aggressiveness increases after delivery^26^,^27^ - a physiological process accompanied with hormonal changes that leads to changes in behavior^47^,^48^ required to acquire and maintain dominant status. Indeed, we found that after delivery, submissiveness markedly decreased in Sub dams compared to nulliparous Sub females (Fig. 6b), correlating with a reduction in Syn IIb expression (Fig. 6c). The impact of metabolic changes related to lactation may potentially increase motivation to compete for access to the feeder, which should be further evaluated.

Another important element underlying social interaction is sexual behavior (mating), known to induce anxiolytic^35^ and antidepressant^36^ effects in males. We showed here that in Sub males, mating is associated with marked reduction of Syn IIb expression, in agreement with reduction of their submissive behavior (Fig. 7).

The importance of studying the regulatory mechanisms of submissiveness stems from its critical role in behavioral abnormalities such as depressive-like and personality disorders^3^,^11^,^49^. The ability of antidepressants to reduce submissive behavior was clearly demonstrated by different research groups^4^,^50^,^51^. Paroxetine, a member of the SSRI class of antidepressants, not only decreased submissiveness of Sub mice but also downregulated expression of Syn IIb in the hippocampus of these mice (Fig. 8). These results agree well with our previous studies showing dose-dependent antidepressant-like effect of paroxetine upon Sub mice in the Forced Swim Test (FST)^2^. Finally, the marked upregulation of Syn IIb observed in Sub pups since the postnatal day 1 (Fig. 4) underlines the link between submissive phenotype and Syn IIb expression, and suggests inheritability of animals’ submissive features^2^.

Given the role of Syn IIb in synaptic activity, this work sheds light on the changes in synaptic plasticity influencing social behavior^52^,^53^, supported by electrophysiological study demonstrating differences in synaptic plasticity among Dom and Sub mice^54^. Presently, by employing different behavioral strategies, we clearly demonstrate that Syn IIb plays an important role in regulation of submissive behavior. Accumulating evidence underlines the importance of social interactions in the etiology of various pathological conditions such as depression, personality disorders and neurodegenerative diseases^6^,^7^,^8^. Further study of Syn IIb as a key regulator of social behavior will highlight its potential as a therapeutic target or biomarker of treatment response.

## Material and Methods

### Animals

The populations of dominant (Dom) and submissive (Sub) mice used in this study were selectively bred on basis of their behavior in the Dominant–Submissive Relationship (DSR) test^2^,^11^. These animals are descendants of the outbred Sabra strain, which freely develop relationships of dominance and submissiveness in the DSR test and whose behavioral and biochemical characteristics were recently found to lie within the range of those of C57BL/6, Balb/c and ICR mice^55^. Outbred Sabra mice were used as a wild type (WT) control group. Animals were housed 5 per cage, given standard laboratory chow and water *ad libitum*. During DSR testing, chow was provided according to the DSR protocol^2^,^11^; see also description below. The colony room was maintained on a 12 h L:12 h D cycle (lights on 07:00–19:00 h). The experiments were conducted in compliance with NIH/USDA guidelines, under the approval of the Ariel University Animal Care and Use Committee (study approval number IL-41-11-12). After experiments WT, Dom and Sub males and females (dams on day 8 after the delivery, and nulliparous (NP) females) were anesthetized in a CO_2_ chamber and decapitated immediately afterwards. Brain regions of interest were dissected, frozen immediately in liquid nitrogen, and stored for future use in a −80 °C freezer.

### Dominant–Submissive Relationship (DSR) test

DSR test was done as described previously^2^,^11^. The DSR apparatus, made from Plexiglas, consists of two identical chambers (12 × 8.5 × 7 cm) joined by a tunnel (2.5 × 2.5 × 27 cm) with a 0.5 cm diameter hole in its bottom center. A self-refilling feeder is connected to the tunnel, allowing a constant supply of sweetened milk (3% fat, 10% sugar), to which only one animal has access at any given moment. The tunnel has narrow slits cut on both sides of the feeder for easy gate insertion and removal. In this way, the paired mice have an equal starting position at the beginning of each session. The description and schematic presentation of DSR apparatus was presented in detail previously^11^. DSR tests were carried out for four consecutive days. During each 14 h period preceding testing, the mice were deprived of food; water was provided *ad libitum*. Pairs of 8 weeks old mice of the same gender from different home cages were matched for relatively similar weight (average weight 43.7 ± 2.1 g) and were tested according to the DSR protocol daily. During each 5 minute DSR session, milk drinking time was recorded manually.

### Resident–Intruder test

The Resident-Intruder test was performed as previously^2^. Briefly, cages were divided into two identical compartments (18 cm × 20 cm) by a transparent divider. 12 weeks old males previously exposed to the DSR test were used in this experiment. A resident male was placed in one of the compartments for a 30 min habituation period, after which an intruder was placed in the neighboring compartment. 10 min later the divider was removed and animals (resident and intruder) were physically exposed to each other for 10 min. Time each animal engaged in aggressive behavior (chasing, biting or scratching) during this time was recorded.

### Microarray analysis

Microarray analysis of mRNA extracted from the hippocampi of WT, Dom and Sub 12 weeks old males previously exposed to the DSR test was conducted using Illumina’s MouseWG-6 v2.0 Expression BeadChip by BioRap Technologies (Rappaport Research Institute, Technion, Israel). Gene expression analysis was performed using the Partek Genomic Suit, using the sentrix number (chip barcode number) as a random factor. Using Principle component analysis (PCA), the chips were clearly different and contributed to random variation, hence this batch effect was corrected. All sample controls appeared similar, hence all remained for the following analysis steps. Of all the 45,000 transcripts on the chips, those represented in all 12 samples below background levels were filtered out, resulting in the removal of about 20,000 transcripts. Also filtered out were those transcripts demonstrating less than 10% variation, yielding a group of approximately 700 transcripts. The two-way analysis of variance (ANOVA) was employed with false discovery rate (FDR) statistical correction for multiple test statistical analysis. The preprocessed data were then clustered using hierarchic cluster analysis with Pearson’s correlation metrics, followed by single-linkage method. Genes were attributed to gene ontology clusters using the DAVID bioinformatics tool^56^,^57^ with the following configuration: kappa similarity: term overlap = 3, similarity threshold = 0.85; at least three members in the group; multiple linkage threshold = 0.5; enrichment threshold, EASE = 1, classification stringency = high; categories: GO-term_BP, GO-term_CC, GO-term_MF; Pathways: KEGG and Biocarta; Protein domains: Interpro, PIR superfamily, SMART; Evaluated: overrepresentation fold change ≥ 2.5; FDR < 1%.

### qRT-PCR analysis

Total RNA was isolated using a 5 Prime Perfect Pure RNA Tissue kit, including a DNAse treatment procedure (5 Prime, USA, Cat. 2302410). Purity, integrity and concentration of the isolated RNA samples were determined by spectrophotometric absorbance at 260 nm. RNA (1 μg) was reverse transcribed in a final volume of 20 μl, using a reverse transcription system (Promega, USA, A3500) with random primers, according to the manufacturer’s instructions. mRNA levels were analyzed by quantitative real time PCR (qRT-PCR) using SYBR Fast Universal Readymix Kit (KAPA, Woburn, MA, USA). Reactions were carried out in the MxPro3000 apparatus (Stratagene, Santa Clara, CA, USA). Primers for tested genes were designed using exon-exon junction principle as follows: Syn IIa (F: 5’ tcctcctcctcttcctcctc 3’, R: 5’ gaagctgaacgcatttgtca 3’); Syn IIb (F: 5’ cagcaagacccctcctcag 3’, R: 5’ aagaagcttggacttgttttgg 3’) and HPRT (F: 5’ tgttgttggatatgcccttg 3’; R: 5’ ttgcgctcatcttaggcttt 3’). HPRT gene was used as an endogenous normalization factor. Primers were synthesized by Integrated DNA technologies (Coralville, IA, USA).

### Sample Preparation for proteomic analysis

Protein extraction from 12 weeks old males previously exposed to the DSR test were performed using Transport Buffer (20 mM Tris/HEPES; 110 mM Potassium acetate; 5 mM Magnesium acetate; 0.5 mM EGTA; 0.1 mM PMSF and 0.1% Triton x100) and titrated with KOH to pH 7.3. Protease and phosphatase inhibitors (0.1 M Sodium vanadate; 0.1 M Sodium tartrate; 0.1 M Sodium molybdate; 0.4 M Fenvalerate, as well as Complete Protease Inhibitor (1:25, Roche, Cat#1838145)) were added. Following homogenization and extraction, 6 M Guanidin-HCl and 105 mM TCEP (dissolved in 25 mM Ammonium bicarbonate) were added to protein solution and incubated for 1 hour at 57 °C. After incubation, 210 mM Iodacetamid (dissolved in 25 mM Ammonium bicarbonate) was added and protein samples underwent additional incubation for 45 min at room temperature, protected from light. Further Guanidine-HCl 6 M was diluted to 1 M with 25 mM Ammonium bicarbonate, after which Trypsin 1:50 was added (with following 1 M Ammonium bicarbonate titration to pH 7.5-8.2). After overnight incubation at 37 °C, sample solutions were adjusted to pH 3 with 10% Formic acid and frozen immediately using liquid nitrogen.

### Liquid Chromatography

ULC/MS grade solvents were used for all chromatographic steps. Each sample was loaded using split-less nano-Ultra Performance Liquid Chromatography (10 kpsi nanoAcquity; Waters, Milford, MA, USA) in high-pH/low-pH reversed phase (RP) 2 dimensional liquid chromatography mode. 20 μg of digested protein from each sample was loaded onto a C18 column (XBridge, 0.3 × 50 mm, 5 μm particles, Waters). The following two buffers were combined: (A) 20 mM Ammonium formate, pH 10 and (B) Acetonitrile (ACN). Peptides were released from the column using a step gradient: 10.8%B, 13.8%B, 15.8%B, 17.8%B, 20.1%B, 23.4%B, 65%B. Each fraction flowed directly to the second dimension of chromatography. The buffers used in the low pH RP were: (A) H_2_O + 0.1% Formic acid and (B) ACN + 0.1% Formic acid. Desalting of samples was performed online using a reverse-phase C18 trapping column (180 μm i.d., 20 mm length, 5 μm particle size, Waters). Then the peptides were separated using a C18 T3 HSS nano-column (75 μm i.d., 200 mm length, 1.8 μm particle size, Waters) run at 0.4 μL/minute. Finally, peptides were eluted from the column and loaded onto the mass spectrometer using the following protocol: 3% to 30%B over 60 min, 30% to 95%B over 5 min, 95% maintained for 7 min (followed by return to initial conditions).

### Mass Spectrometry

The nanoLC was coupled online through a nanoESI emitter (7 cm length, 10 mm tip; New Objective; Woburn, MA, USA) to a quadrupole ion mobility time-of-flight mass spectrometer (Synapt G2 HDMS, Waters) tuned to 20,000 mass resolution (full width at half height). Data were acquired using Masslynx version 4.1 in data independent acquisition mode (DIA), HDMSE positive ion mode. The ions were separated in the T-Wave ion mobility chamber and transferred into the collision cell. Collision energy was alternated from low to high throughout the acquisition time. In low-energy (MS1) scans, the collision energy was set to 5 eV and this was ramped from 27 to 50 eV for high-energy scans. For both scans, the mass range was set to 50–2,000 Da with a scan time set to 1 second. A reference compound (Glu-Fibrinopeptide B; Sigma) was infused continuously for external calibration using a LockSpray and scanned every 30 seconds.

### Data Processing, Searching and Analysis

Raw data processing and database searching was performed using the Proteinlynx Global Server (PLGS) version 2.5.2. Database searching was carried out using the Ion Accounting algorithm^58^. Data were searched against a combined target and reversed (decoy) mouse sequences in UniprotKB database and the CRAP list of common laboratory contaminants, version 2013_06 with 50,901 entries. Trypsin was set as the protease, with two missed cleavages allowed. Carbamidomethylation was set as a fixed modification and oxidation of methionine as a variable modification. Raw data were also imported into Rosetta Elucidator System, version 3.3 (Rosetta Biosoftware, Seattle, WA, USA). Elucidator was used for alignment of raw MS1 data in RT and m/z dimensions as described^59^. Aligned features were extracted and quantitative measurements obtained by integration of three-dimensional volumes (time, m/z, intensity) of each feature as detected in the MS1 scans. Search results were then imported directly from PLGS for annotation and the minimum identification score was set to achieve a maximum global false discovery rate of 1%.

Relative protein abundance was calculated using the Hi-3 method^60^. Preprocessed data were subjected to hierarchic cluster analysis using Euclidean metrics and Ward’s linkage method. Clusters were submitted to one way ANOVA analysis for statistical significance. Cluster number was validated using root mean square standard deviation (RMSSTD) and Dunn’s test analysis. Proteins were attributed to gene ontology analysis using the DAVID bioinformatics platform with parameters as described in microarray data post-processing analysis section.

### Western blot analysis

Protein extracted from 12 weeks old males previously exposed to the DSR test was used for Western blot analysis. Extractions were performed on ice using lysis buffer (20 mM Tris/HEPES pH 7.4-8.0; 10 mM NaCl/KCl; 0.1% Triton x100) with protease and phosphatase inhibitors (Potassium sodium tartrate tetrahydrate 0.1 M; Sodium molybdate 0.1 M; Sodium orthovanadate 0.1 M; Complete protease inhibitor (1:25, Roche, Cat#1838145)) and sonication.

Western blot analysis of Synapsin II isoforms protein expression utilized anti-synapsin II (1:1000; Cat.106203; Synaptic Systems, Goettingen, Germany) and anti-histone H3 (1:10000; (05-928) EMD Millipore Corporation, Billerica, MA, USA) primary antibodies. After hybridization to a horseradish peroxidase-conjugated anti-rabbit secondary antibody (1:3000; #170-6515, Bio-Rad Laboratories, Hercules, CA), and brief incubation with ECL solution (Luminata Crescendo Western HRP substrate, EMD Millipore Corporation, Billerica, MA, USA), blots were visualized (Image Quant LAS4000 mini; GE Healthcare, Milwaukee, WI, USA) and densometrically analyzed using ImageQuant TL software (GE Healthcare). The ratio of each protein band to the H3 band was used for quantitative analysis.

### Statistical analysis

The statistical significance between animal groups was assessed using one- and two-way ANOVA followed by Bonferroni post hoc multiple comparison test (GraphPad Prism version 5.02). Statistical differences are shown as * at *p* < 0.05, ** at *p* < 0.01, and *** at *p* < 0.001.

## Author Contributions

E.N. and A.P. performed study design, data acquisition, analysis and interpretation. T.T., G.G., M.G. and M.B. contributed substantially to molecular and behavioral data acquisition and interpretation. M.S-D. and I.M. contributed substantially to transcriptomic data analysis. Y.L. and I.M. contributed substantially to proteomic data acquisition and analysis. E.N., I.K., M.G., I.M., G.Y. and A.P. wrote the main manuscript text. All authors revised manuscript critically and gave final approval.

## Additional Information

**How to cite this article**: Nesher, E. *et al*. Synapsin IIb as a functional marker of submissive behavior. *Sci. Rep.* **5**, 10287; doi: 10.1038/srep10287 (2015).
